# Carbon nanotube-based, serially connected terahertz sensor with enhanced thermal and optical efficiencies

**DOI:** 10.1080/14686996.2022.2090855

**Published:** 2022-07-05

**Authors:** Daichi Suzuki, Yuma Takida, Yukio Kawano, Hiroaki Minamide, Nao Terasaki

**Affiliations:** a Sensing System Research Center, National Institute of Advanced Industrial Science and Technology (AIST), Saga, Japan; b RIKEN Center for Advanced Photonics, RIKEN, Miyagi, Japan; c Faculty of Science and Engineering, Chuo University, Tokyo, Japan; d Laboratory for Future Interdisciplinary Research of Science and Technology, Tokyo Institute of Technology, Tokyo, Japan; e National Institute of Informatics, Tokyo, Japan

**Keywords:** Carbon nanotube, terahertz, photo-thermo-electric effect, optical antenna, laser ablation, spectroscopy

## Abstract

Owing to their high thermal and optical performances, carbon nanotube (CNT) films are used in various photo-thermo-electric (PTE) applications, such as terahertz (THz) sensing and energy harvesting. To improve the performance of PTE devices, a device structure should be designed based on a deep understanding of the thermal and optical responses of the CNT film. However, the optical properties of CNT films in the THz frequency region remain unclear because of the difficulties associated with device processing and measurements. Herein, we report our findings on the thermal and optical characteristics of CNT films. The shape of the CNT film that maximizes the product of the thermal and optical factors (optimal structure of the PTE sensor) depends on the frequency of the irradiating electromagnetic wave. The optimal film thickness and width values for THz irradiation range from 300–600 nm and 50–70 µm, respectively. Subsequently, we fabricated a serially connected, multi-element PTE sensor with an optimal device structure and enhanced the detection sensitivity by approximately 13 times compared with a single-element PTE sensor. In addition, we demonstrated the first THz spectroscopy application using a PTE sensor. The findings of this study, thermal/optical factor enhancement, and micro-sized CNT film processing technology can be used to improve the performance of all CNT-based photothermal devices, including PTE sensors and thermoelectric generators.

## GRAPHICAL ABSTRACT

**Figure:**
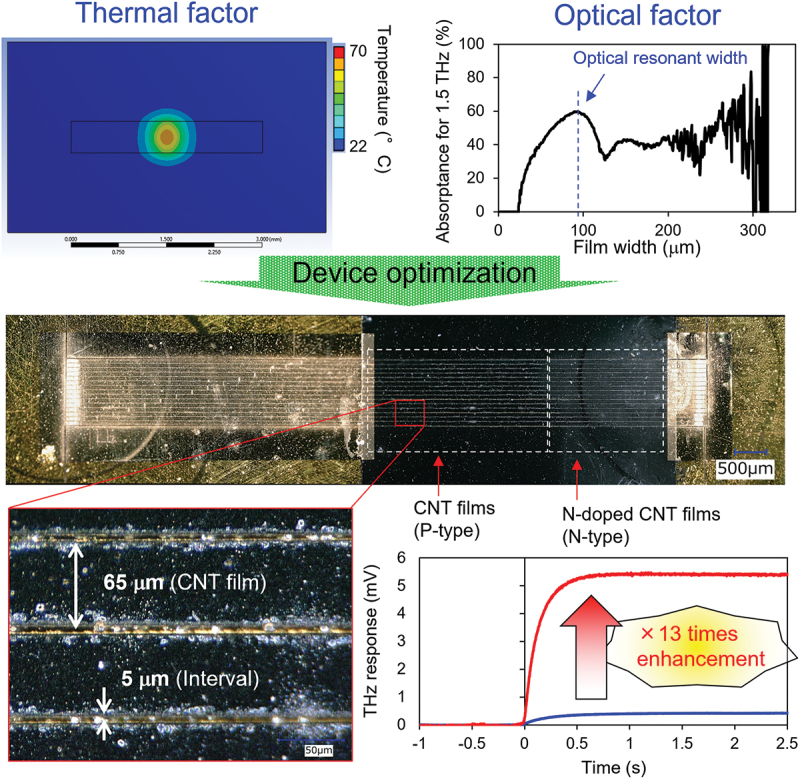

## 1. Introduction

Sensing systems based on electromagnetic wave technology have been used extensively in various fields. Organ diagnosis using ultrasonic waves, traffic congestion measurement using laser reflection, and baggage inspection using X-rays at airports are a few practical examples of sensing systems based on the inherent nature of electromagnetic waves, such as transmission, reflection, and diffraction [1–5]. Among them, the technology based on terahertz (THz) frequency waves is gaining popularity as a powerful nondestructive sensing tool owing to its unique capabilities of high transparency for soft materials and resonant response with intermolecular vibration [6–8]. Various studies have been conducted on the practical use of THz sensing, including material science [9–12], component fabrication (such as emitters and detectors) [13–15], system development [16,17], and sensing applications [18,19]. In the case of component fabrication, the challenge is to create a structure that efficiently couples with a THz wave, having a rapid cycle time of sub-picoseconds and small photon energy amounting to several meV. Thus, low-dimensional and metamaterial structures are often utilized [20–22]. Carbon nanotubes (CNTs) have wide-band absorption characteristics from sub-THz to near-infrared frequencies owing to the plasmon resonance in the longitudinal direction of the nanotube [23,24]. Furthermore, CNTs exhibit high photo-thermo-electric (PTE) conversion efficiency derived from the low-dimensional structure [25,26]. Therefore, CNTs are expected to serve as potential materials for THz components. Using the PTE effect in CNT films, we previously developed PTE sensors, scanners, and cameras that work in a broad frequency range from sub-THz to near-infrared and demonstrated nondestructive sensing applications [27–35]. Assuming a system that does not dissipate heat to the surrounding, the PTE voltage ΔV can be expressed as shown in Equation (1). Thus, we have

**Figure 1.**
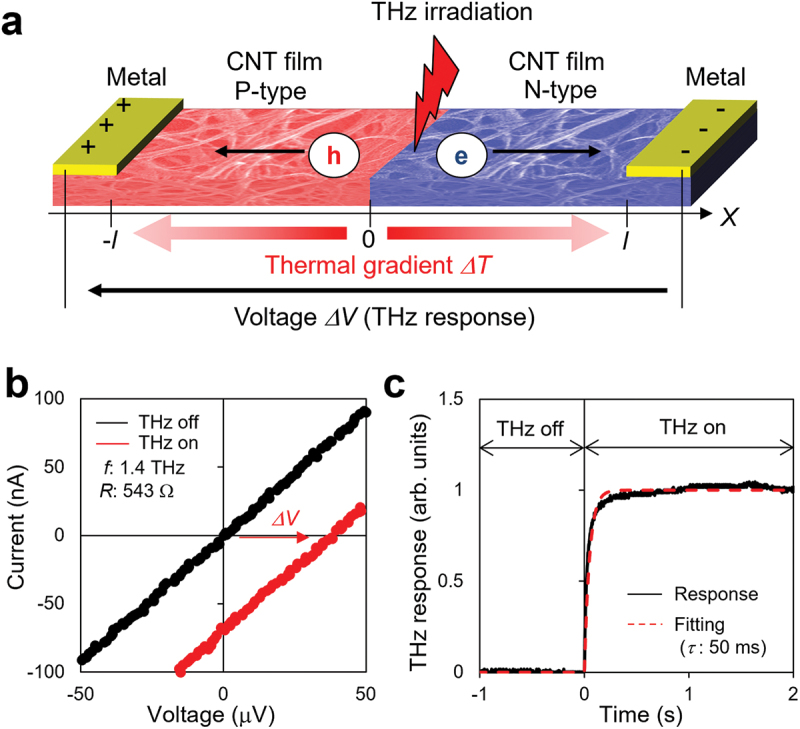
(a) Schematic of the photo-thermo-electric (PTE) sensor. The mechanism of operation is based on the following three steps: 1) conversion of light to heat, 2) diffusion of electrons and holes along a thermal gradient, and 3) generation of voltage; (b) Typical I-V curve of the PTE sensor. Both the thermal and optical factors govern the PTE voltage, as indicated in Equation (1) and (2); (c) Typical transient response of the PTE sensor. The black solid line indicates the experimental THz response and the red dashed line indicates the fitting curve with the transient function of 1 - e^−t/τ^, whereby τ is the time constant.

(1) ΔV=S×ΔT

and

(2) $$\Delta T=\overset{l}{\underset{x=0}{\int }}R\left(x\right)Q_{absorb}dx\approx \frac{l}{k\times t\times w}\times Q_{in}\times \alpha \;\;,$$

where *S* is the Seebeck coefficient, *T* is the temperature, *R* is the thermal resistance, *Q*_in_ is the power of THz irradiation, and *Q*_absorb_ is the absorbed power in the CNT film. Additionally, *k*, *l*, *t*, *w*, and *α* are the thermal conductivity, length, thickness, width, and THz absorption coefficient of the CNT film, respectively. Based on Equation (2), the PTE effect is governed by both the thermal (the first term on the right-hand side of Equation (2) and optical factors (the third term on the right-hand side of Equation (2). The schematic and typical response of a PTE sensor is shown in Figure 1. According to steady-state thermal simulations conducted to design the thermal factors, we observed that the THz response (PTE voltage) can be enhanced by reducing the total heat capacity; this can be achieved by reducing the thickness and width of the CNT film from 200 to 2 µm and 6000 to 500 µm, respectively [35]. In our previous studies, a minimum thickness of 2 µm and width of 500 µm for the CNT film was sufficiently large to absorb THz waves; therefore, we focused only on the enhancement of thermal efficiency to achieve higher sensitivity [35]. However, the findings of previous studies, that is increasing the thermal factors and sensitivity by downsizing the device, are only valid on the millimeter or micrometer scale. This is because, if the CNT film is miniaturized to nanometer or angstrom scales to increase the element density, the absorption efficiency for THz waves drastically decreases to zero owing to the diffraction limit, and thus, the sensitivity deteriorates. To further improve the device performance by enhancing the photo-thermal efficiency of the CNT film by downsizing it to a micrometer scale or less, an analysis should be performed that considers the thermal and optical factors in detail. However, the optical characteristics in the THz frequency region of the CNT film have rarely been studied; this is primarily attributed to the difficulties associated with processing the CNT film. To acquire the THz spectrum of a CNT film, a CNT film should be processed with sufficient thickness (>1 µm) and a spatial resolution equal to half the wavelength of the THz wave (>10 µm). This cannot be fabricated by conventional lithography or inkjet processes because of the high aspect ratio of the thickness and microscale spatial resolution of the CNT film [36,37]. Therefore, studies on CNT film processing and analysis of both thermal and optical factors is a critical issue to enhance PTE device performance.

Therefore, in this study, we report the results of our investigation on the structures of PTE devices with enhanced thermal and optical efficiencies. By employing the original laser ablation method [38], we fabricated the first CNT film-based THz antennas that cannot be fabricated by conventional lithography or inkjet processes and revealed the optical characteristics in the THz frequency region of the CNT film. In addition, we validated the device structure in which the product of the thermal and optical factors is optimal through both steady-state thermal simulation and optical spectroscopic measurements. The laser ablation method is advantageous in that even thick CNT films can be cut at microscale spatial resolution. This enables us to array single-element sensors in series at high densities. Consequently, the sensitivity was improved by 13 times through optimization and integration of the device structure. Correspondingly, the first THz spectroscopy application using the PTE sensor was demonstrated. We expect that the thermodynamic and optical structure optimization for CNT films, which is the result of this study, will be useful not only for THz sensing but also for a variety of fields, such as thermoelectric power generation and material development.

## 2. Experimental section

### 2.1. Materials and components

**Figure 2.**
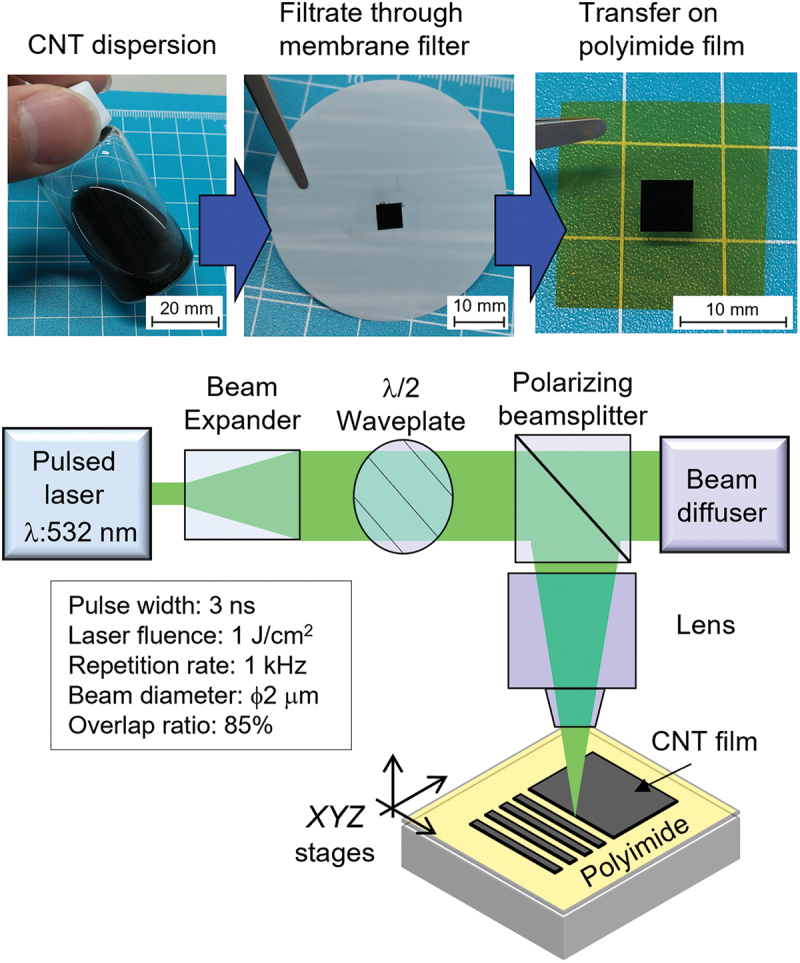
Photographs of the carbon nanotube (CNT) film and schematic of the laser ablation setup.

The CNT dispersion was purchased from ZEON Corporation. The physical properties of the CNTs were as follows: super-growth single-walled, metallic-semiconducting mixed, electrical conductivity of 500 S cm^−1^, and a Seebeck coefficient of 60 µV K^−1^ (pristine) and −60 µV K^−1^ (*n*-doped). The CNT dispersion was filtered through polyvinylidene difluoride membrane filters (thickness: 125 µm; pore size: 0.1 µm) and subsequently transferred onto a polyimide film. The thickness of the CNT film was measured using confocal laser microscopy (VK-X1000/1050, KEYENCE CORPORATION) (see the Supplemental material for the height profile of the CNT film). The CNT film was processed into various shapes (device and antenna) using the laser ablation method (Figure 2) [38]. The laser setup was as follows: the pulse width, wavelength, numerical aperture of the objective lenses, repetition rate, fluence, and overlap ratio were 3 ns, 532 nm, 0.3, 1 kHz, 1 J cm^−2^, and 85%, respectively. The n-doping solution (a mixture of potassium hydroxide and benzo-18-crown-6-ether, 0.5 mol L^−1^) was used to form a p-n junction in the CNT film [39]. Gold (Au) lead electrodes were evaporated using a vacuum evaporator. THz waves were irradiated at the p-n junction of the CNT film and the PTE voltage was measured using a multimeter. We prepared three types of emitters: a far-infrared laser (FIRL-100, Edinburgh Instruments Ltd.) for 1.4 THz illumination, an injection-seeded terahertz parametric generator (is-TPG) for 1–2.8 THz illumination (see the Supplemental material for the system and power spectrum) [40–42], and an infrared light-emitting diode (L14336–0083 R, Hamamatsu Photonics K.K.) for 361 THz illumination.

### 2.2. Steady-state thermal distribution

To clarify the thermal factor of the device structure, we performed steady-state thermal distribution of the devices via the finite element method using ANSYS software. Thermal distribution was calculated based on the solution of the following heat equation:

(3) $$\rho c\frac{\partial T}{\partial t}=k\left(\frac{\partial^{2}}{\partial x^{2}}+\frac{\partial^{2}}{\partial y^{2}}+\frac{\partial^{2}}{\partial z^{2}}\right)T+Q\;\;,$$

where *ρ* is the density, *c* is the specific heat capacity, *k* is the thermal conductivity, and *Q* is the total heat [35]. The steady-state thermal distribution was calculated by solving Equation (3), subject to the condition ∂T/∂t = 0 (steady-state). In this study, we assumed that the light absorption rate was 100% and that heat was applied directly onto the surface of the CNT film to focus only on the thermal factor. The physical parameters of materials were as follows: the thermal conductivities of the X-Y plane of the CNT film, Z-axis of the CNT film, Au electrodes, and polyimide film were 10 W m^−1^ K^−1^, 0.1 W m^−1^ K^−1^, 315 W m^−1^ K^−1^, and 0.16 W m^−1^ K^−1^, respectively, and the heat transfer coefficient of air was 4.65 W m^−2^ K^−1^ in still conditions at 300 K (see the Supplemental material for details of the simulation model).

### 2.3. Spectroscopic measurements

To clarify the optical factor of the device structure, we performed THz time-domain spectroscopy (THz-TDS) (TAS7500TS, Advantest Corporation) in a transmission measurement configuration [43]. The measurement conditions were as follows: data averaging ratio: 1028 times, frequency bandwidth: 0.5–4.5 THz, temporal resolution: 2 fs, frequency resolution: 3.8 GHz, scan range: 262 ps, throughput: 16 ms/scan, frequency accuracy: 10 GHz (at 1.41 THz), and a spot diameter of 0.5 mm for THz irradiation. All the systems included samples and the light passages were filled with dry air to avoid undesired THz absorption by water vapor.

## 3. Results and discussion

### 3.1. Thermal and optical characteristics of CNT films with respect to film thickness

**Figure 3.**
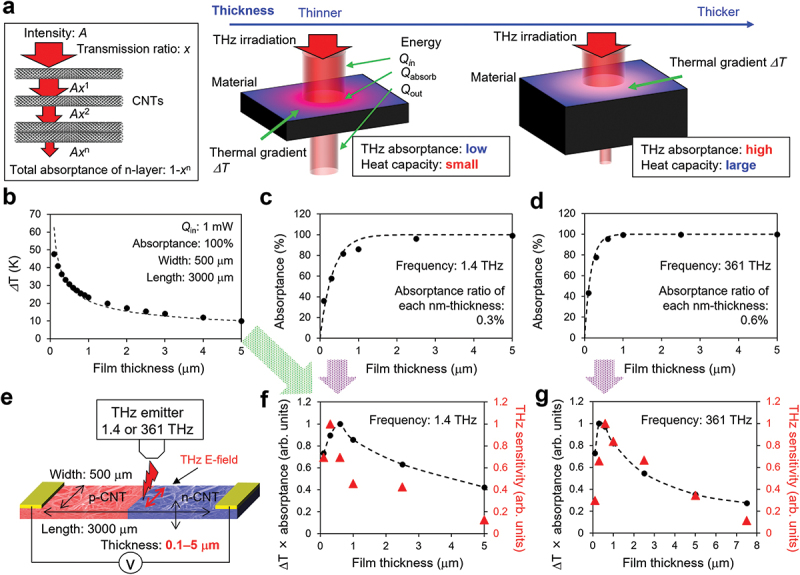
Thermal and optical characteristics of CNT films with respect to the film thickness. (a) Conceptual schematic of the relationship between the thermal/optical factors and film thickness; (b) Thermal characteristics along the film thickness; optical characteristics for (c) 1.4 THz and (d) 361 THz irradiation along the film thickness; (e) Experimental setup of THz response; product of the thermal and optical factors (black dots) and experimental results of THz sensitivity (red triangles) for (f) 1.4 THz and (g) 361 THz irradiation.

According to Equation (2), the thermal factor of the CNT film is expected to be inversely proportional to film thickness. The optical factor along the film thickness can be calculated using the simple model shown in Figure 3(a) as 1 - *x*^t^, where *x* is the transmission ratio for each CNT film thickness, indicating that the optical factor decreases with thinner CNT films. Therefore, we investigated the thermal and optical characteristics of CNT films with respect to film thickness to determine the film thickness at which both factors were optimal. We prepared several types of devices with *w* = 500 µm, *l* = 3000 µm, and *t* = 0.1–5 µm, where the film thickness was controlled by tuning the filtration amount of CNT dispersion (see the Supplemental material). Figure 3(b) shows the simulation results of the thermal characteristics of CNT films with respect to film thickness. The thermal distribution agrees with the concept that the thermal factor is enhanced with thinner films. Figure 3(c,d) show the optical characteristics of 1.4 THz and 361 THz of the CNT films with respect to film thickness obtained from the experimental results; the results indicate that the optical factor deteriorates when thinner films are used. These results were fitted using an absorbance ratio of 0.3% for 1.4 THz CNT films and 0.6% for 361 THz CNT films. The products of the thermal and optical factors are shown in Figure 3(f,g) (black dots) and the film with a thickness of approximately 300–600 nm can maximize the product of the thermal and optical factors. The experimental results of THz sensitivity shown in Figure 3(f,g) (red triangles) represent the same behavior, wherein the sensitivity for both 1.4 THz and 361 THz irradiation was maximized for a film thickness of approximately 300–600 nm. Based on these results, we inferred that the optimum CNT film thickness for the entire THz frequency region ranged from 300–600 nm.

### 3.2. Thermal and optical characteristics of CNT films with respect to the film width

**Figure 4.**
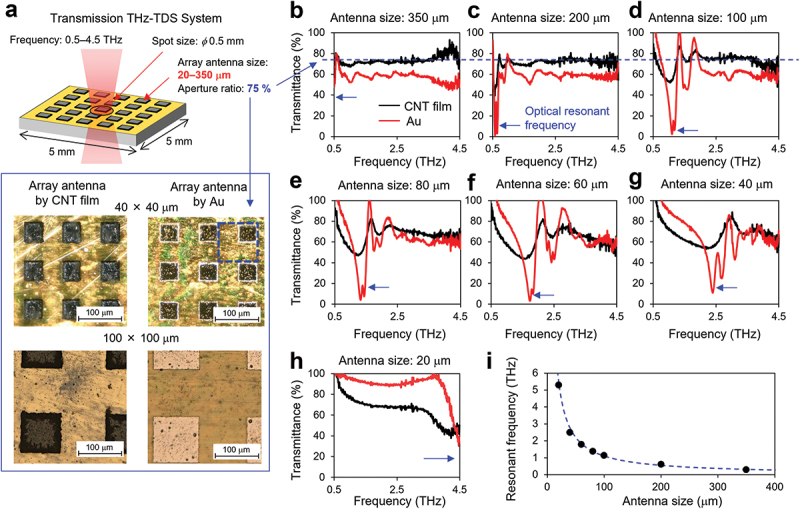
(a) Schematics of antennas and measurement system of the THz time-domain spectroscopy (THz-TDS); transmittance spectrum of the antennas with the antenna size of (b) 350 × 350 µm, (c) 200 × 200 µm, (d) 100 × 100 µm, (e) 80 × 80 µm, (f) 60 × 60 µm, (g) 40 × 40 µm, and (h) 20 × 20 µm. The blue arrows indicate the optical resonant frequency; (i) Resonant frequency versus antenna sizes.

Subsequently, we investigated the thermal and optical characteristics of CNT films with respect to the film width. We observed that the thermal factor behaves similarly with respect to film width as it does with respect to film thickness (Equation (2)); in contrast, the optical factor along the film width is more complicated owing to optical resonance and diffraction. For general bulk materials, such as Au, these phenomena depend on the relationship between the material size and wavelength of the irradiated electromagnetic wave [44]. Although the optimum value of CNT film thickness that maximizes the optical factor is expected to change depending on the frequency of irradiation in general bulk materials, the optical characteristics of the CNT film in the THz frequency region have not been extensively studied owing to difficulties in antenna fabrication, as described in the introduction. Therefore, in this study, we utilized the original laser ablation method [38] to fabricate THz antennas using CNT films and measured the resonant spectrum of the CNT film to determine their optical characteristics in the THz frequency region. Figure 4 shows the schematic of the transmission THz-TDS system. The CNT film and Au (reference) array antennas were formed on a polyimide film using the laser ablation method. The chosen dimensions of the antenna ranged from 20 × 20 µm to 350 × 350 µm to match the half-wavelength wave range of 0.5–5 THz; the CNT film thickness was 1.4 µm and the aperture ratio was designed to be 75%. Figure 4(b–h) show the transmittance spectrum of different antenna sizes and reveal the following four features of the optical characteristics of the CNT film: a) Optical resonance occurs when the antenna size is approximately half the wavelength of the irradiation wave and the absorption rate is maximized. This phenomenon indicates that the resonant frequency can be controlled freely by tuning the film width, as shown in Figure 4(i); b) In the frequency region wherein the size of the antenna is larger than half the wavelength of the irradiation wave, the antenna exhibits a constant transmittance response depending on its aperture ratio (75% in this case); c) In the frequency region wherein the antenna is smaller than half the wavelength of the irradiation wave, the absorption rate drops abruptly as the frequency decreases; d) The CNT film and Au individually exhibit similar behaviors; however, the CNT/Au antenna exhibits a broad/sharp resonance. This implies that the CNT film operates as a wideband sensor with less pronounced frequency dependence. From the aforementioned spectroscopic experiments, it was clarified that an individual optimum film width exists that depends on the frequency of the emitter and maximizes the optical factor of the PTE sensor.

**Figure 5.**
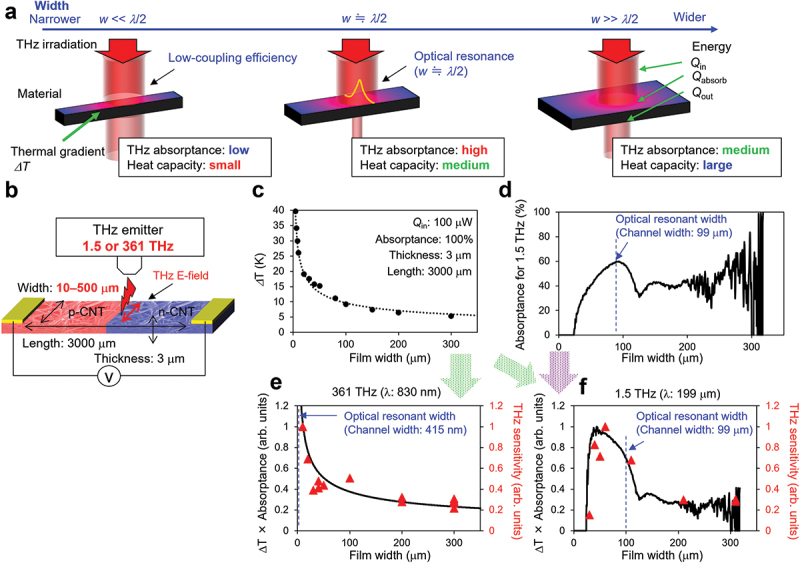
Thermal and optical characteristics of CNT films with respect to film width. (a) Conceptual schematics showing the relationship between the thermal/optical factors and film width; (b) Experimental setup of THz response; (c) Thermal and (d) optical characteristics for 1.5 THz irradiation along the direction of the film width; the product of thermal and optical factors (black line) and experimental results of THz sensitivity (red triangles) for irradiations at (e) 361 THz and (f) 1.5 THz.

Based on the experimental results of thermal and optical factors, we also investigated the film width that maximized the product of the two factors. Figure 5(a,b) show the conceptual image and experimental setup, respectively. We prepared several types of devices with *l* = 3000 µm, *t* = 3 µm, and *w* = 10–500 µm, wherein the film width was controlled using the laser ablation method. Electromagnetic waves at two frequencies (1.5 and 361 THz) were irradiated to understand the frequency dependence of the optical factors. Figure 5(c–f) show the simulation and experimental results of the thermal and optical characteristics of the CNT films with respect to the film width. The thermal gradient ΔT (Figure 5(c)) increased when the film width narrowed. A similar response pattern was obtained for a change in the film thickness (Figure 3(c)). Figure 5(d) shows the film width dependence of the absorbance for 1.5 THz that was measured by the THz-TDS system. Figure 5(e,f) show the plots of the product of the thermal and optical factors (black line) and the experimental THz sensitivity results (red triangles) for irradiations at 361 THz and 1.5 THz, respectively. When the frequency of the irradiated wave was 361 THz, the optical factor of the CNT film was constant because the film width (ranging from 10–500 µm) was much larger as compared with the half wavelength of 361 THz (830 nm). Therefore, as the width of the film narrows, the sensitivity increases because it depends only on the thermal factor (Figure 5(e)). In contrast, in the case of a 1.5 THz irradiation, the optical factor was maximized by the optical resonance at a film width of 99 µm (half wavelength of 1.5 THz). When the film width was smaller than that, the optical factor deteriorated abruptly to zero. Therefore, unlike the irradiation at 361 THz, the product of the thermal and optical factors yielded a peak for the irradiation at 1.5 THz when the film width was approximately 50 µm. Based on the experimental results (red triangles in Figure 5(f)), the sensitivity was maximum when the film width was 60 µm. This agreed with our calculations and thus supported the validity of this research. From the results of this study, we confirmed the importance of designing the CNT film width to an appropriate size according to the frequency of the target light source (for example, approximately 50–70 µm in the case of 1.5 THz) to increase the thermal and optical factors.

### 3.3. Serially connected, multi-element PTE sensor

**Figure 6.**
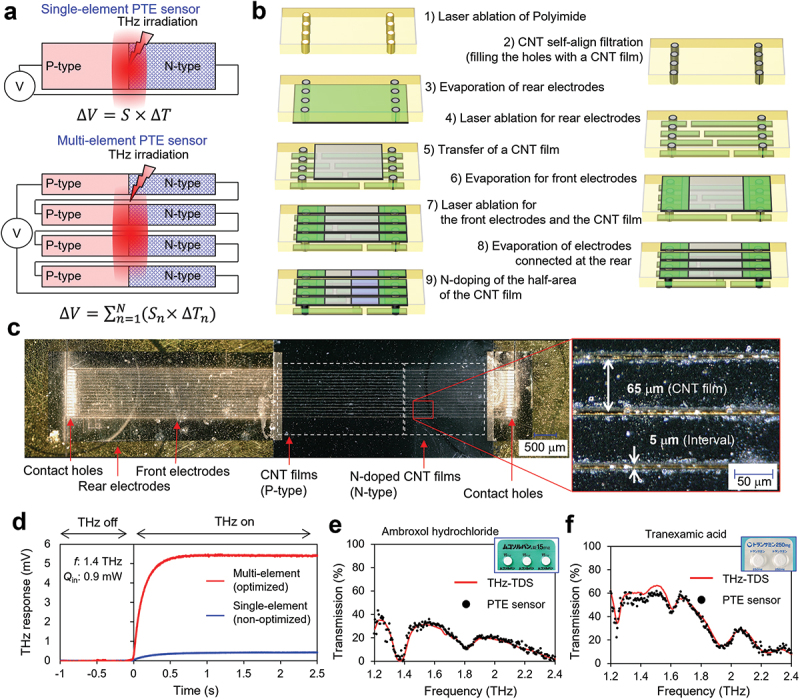
(a) Conceptual schematics of the multi-element PTE sensor; (b) Schematic of the fabrication process; (c) Photographs of the multi-element PTE sensor; (d) THz response comparison between the optimized and non-optimized PTE sensors; THz spectra of (e) ambroxol hydrochloride and (f) tranexamic acid acquired by the multi-element PTE sensor (black dots) and the THz-TDS system (red line).

In this study, we confirmed that the sensitivity of the PTE sensor can be improved by reducing the width and thickness of the CNT film to an appropriate size. Meanwhile, a multi-element structure, in which single-element sensors comprising a pair of *p*-n junctions are arrayed in series, is effective to improve the total PTE voltage, as shown in Figure 6(a) (the structure is similar to that of a general thermopile) [45–47]. Therefore, reducing the size of the sensor improves the sensitivity of a single element and the total PTE voltage by increasing the density of a serially connected, multi-element array. By incorporating all the techniques mentioned in this study, we fabricated a multi-element, serially connected PTE sensor. Figure 6(b,c) show the schematic of the fabrication process and a photographic image of the multi-element PTE sensor, respectively. The sensor was fabricated according to the following steps: 1) laser ablation of a polyimide film, 2) filling the holes with a CNT film via a self-aligned filtration process, 3) evaporation of rear electrodes, 4) laser ablation of rear electrodes, 5) transfer of the CNT film onto the front side of the polyimide film, 6) evaporation of the front electrodes, 7) laser ablation of the front electrodes and CNT film, 8) evaporation of electrodes connected at the rear, and 9) n-doping of half-area of the CNT film to form a *p*-n junction. The thickness and width of the CNT films were 500 nm and 65 µm (optimized size at approximately 1.5 THz according to Figures 3(f) and 5(f)), respectively. The elements were placed at intervals of 5 µm and connected in series via contact holes on both sides of the film. A comparison between the THz response of optimized and non-optimized PTE sensors is shown in Figure 6(d), wherein the thickness and width of the non-optimized PTE sensors were 5 µm and 1 mm, respectively. Owing to the optimization of the film thickness, width, and the number of elements, the sensitivity of the multi-element PTE sensor was improved to 5.9 V W^−1^, which is 13 times higher than that of the single-element PTE sensor (0.46 V W^−1^). The THz spectroscopy of medications (Figure 6(e,f)) was conducted with an is-TPG source to demonstrate the increase in sensitivity. In this experiment, the signal amplitude from the multi-element PTE sensor was measured as a function of the THz-wave frequency of the is-TPG source. The results represent a unique absorption spectrum derived from the vibration of the molecular structure and thus enabled us to distinguish between each medication type (ambroxol hydrochloride and tranexamic acid). To the best of our knowledge, THz spectroscopy using PTE sensors has not been reported before; therefore, we believe that the achievement of THz spectroscopy with a compact and flexible CNT film PTE sensor that does not require a large-scale optical system, such as the THz-TDS system, will contribute to the expansion of the field of THz sensing.

## 4. Conclusion

We studied the thermodynamics and optical characteristics of CNT films using thermal simulations and optical experiments. By analyzing the photo-thermal characteristics of an antenna-shaped CNT film processed using the original laser ablation method, we observed that an optimum size exists for the miniaturization of the thickness and width of the CNT film. Using the findings and techniques of this study, that is, by optimizing the shape according to the irradiation frequency, we improved the sensitivity of the PTE sensor by a factor of 13 and achieved THz spectroscopy. The results of THz sensing constitute a paradigm that can be improved by enhancing the thermal and optical factors. Nanocarbon devices based on the operating principle of heat and/or light are not limited to PTE sensors (THz sensing), and studies on many applications, such as thermoelectric generators, thermal sensors, motion sensors, capacitors, and optical antennas, are underway [46–52]. The thermal/optical factor enhancement and micro-sized CNT film processing technologies presented in this study can be used to improve the performance of the aforementioned applications. Accordingly, we anticipated that our results will play a crucial role in advancing current technologies for both THz sensing and nanocarbon devices.

## Supplementary Material

Supplemental Material
