# Supplementary material for: Carbon nanotube-based, serially connected terahertz sensor with enhanced thermal and optical efficiencies
Source: Sci Technol Adv Mater. 2022 Jul 5;23(1):424–33. doi: 10.1080/14686996.2022.2090855 (PMC9258063; doi:10.1080/14686996.2022.2090855)

Supplemental material

**Carbon nanotube-based, serially connected terahertz sensor with enhanced thermal and optical efficiencies**

Daichi Suzuki^a^*, Yuma Takida^b^, Yukio Kawano^c–e^, Hiroaki Minamide^b^ and Nao Terasaki^a^

*^a^Sensing System Research Center, National Institute of Advanced Industrial Science and Technology (AIST), Saga, Japan; ^b^RIKEN Center for Advanced Photonics, RIKEN, Miyagi, Japan; ^c^Faculty of Science and Engineering, Chuo University, Tokyo, Japan; ^d^Laboratory for Future Interdisciplinary Research of Science and Technology, Tokyo Institute of Technology, Tokyo, Japan; ^e^National Institute of Informatics, Tokyo, Japan*

*E-mail: daichi.suzuki@aist.go.jp

**Fig. S1:** Photographs of the carbon nanotube (CNT) film and Au array antennas. The antenna sizes were designed using the laser ablation method and ranged from 20 × 20 μm to 350 × 350 μm to match the half-wavelength value of 0.5–5 THz waves.


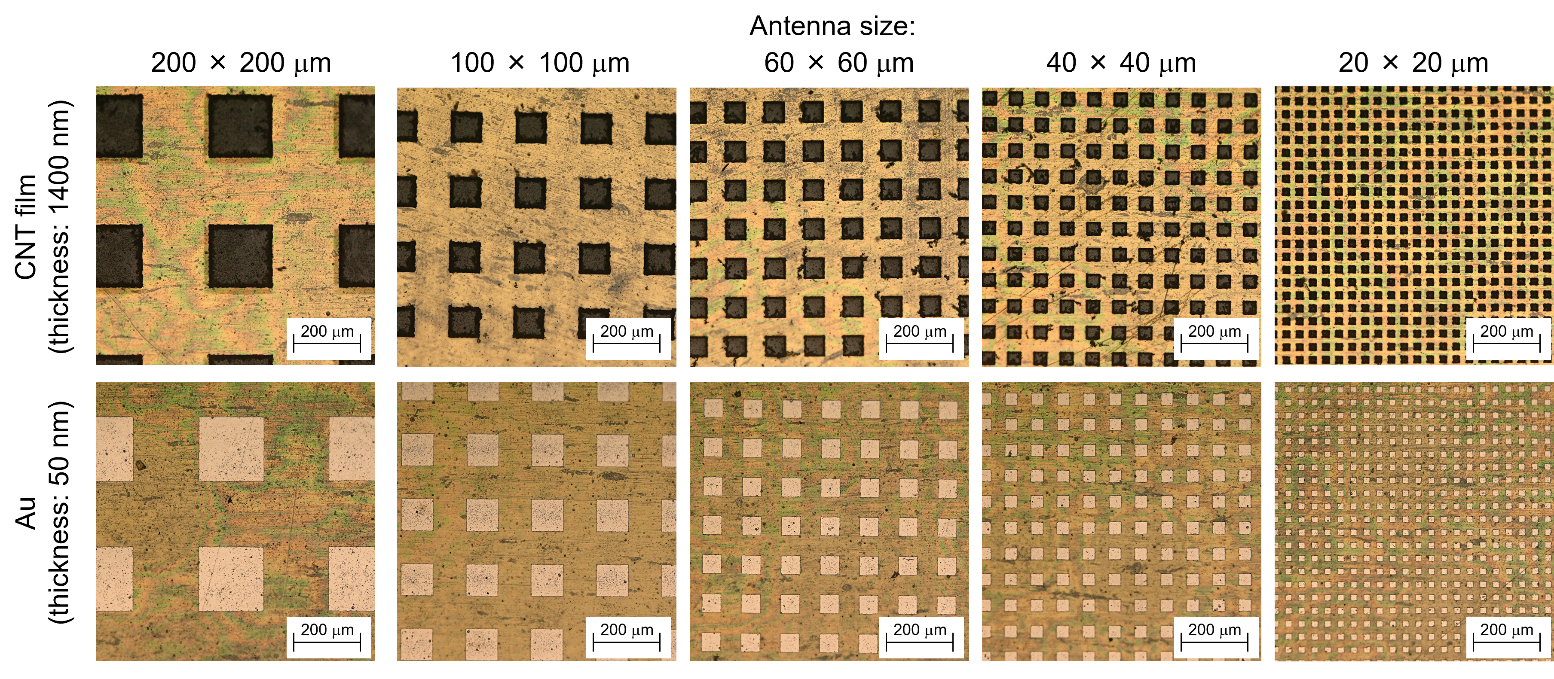


**Fig. S2:** (a) Height profile of the CNT film with the filtration amount of 150 μL; (b) Film thickness versus filtration amount of CNT dispersion. The filtration rate of the CNT film thickness is 4 nm/μL.


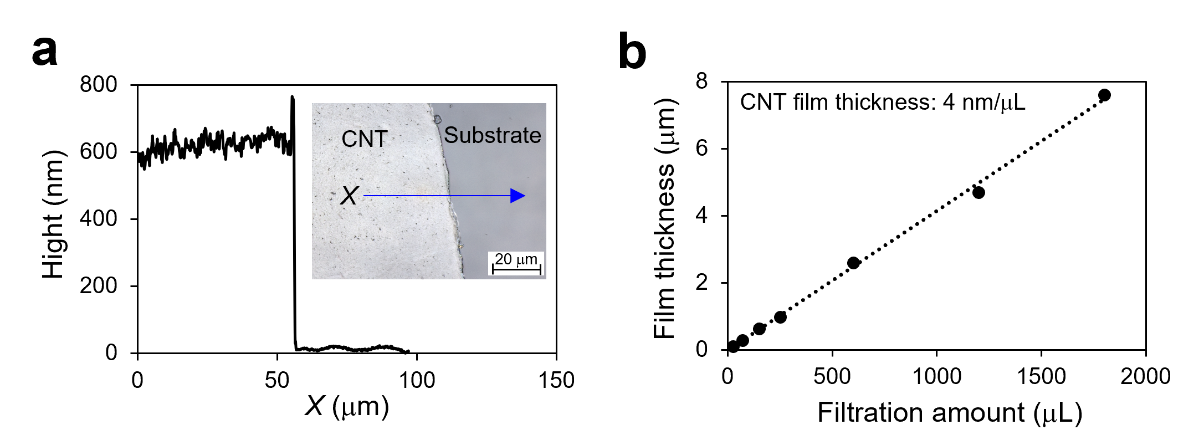


**Fig. S3:** Schematic of the terahertz time-domain spectroscopy (THz-TDS) system. The 0.5­–4.5 THz pulse wave was emitted from a Cherenkov-type THz wave generator using a nonlinear optical crystal (LiNbO_3_) waveguide and was used to irradiate a sample. The transmitted pulse was guided and focused onto the photoconductive antenna (detector) mounted on a hyper-hemispherical silicon lens. The transmittance spectrum was measured under the following conditions: a temporal resolution of 2 fs, frequency resolution of 3.8 GHz, scan range of 262 ps, throughput of 16 ms/scan, frequency accuracy of 10 GHz (at 1.41 THz), and spot diameter of THz illumination of 0.5 mm. All the systems included samples, and light passes were filled with dry air to avoid undesired THz absorption by water vapor.


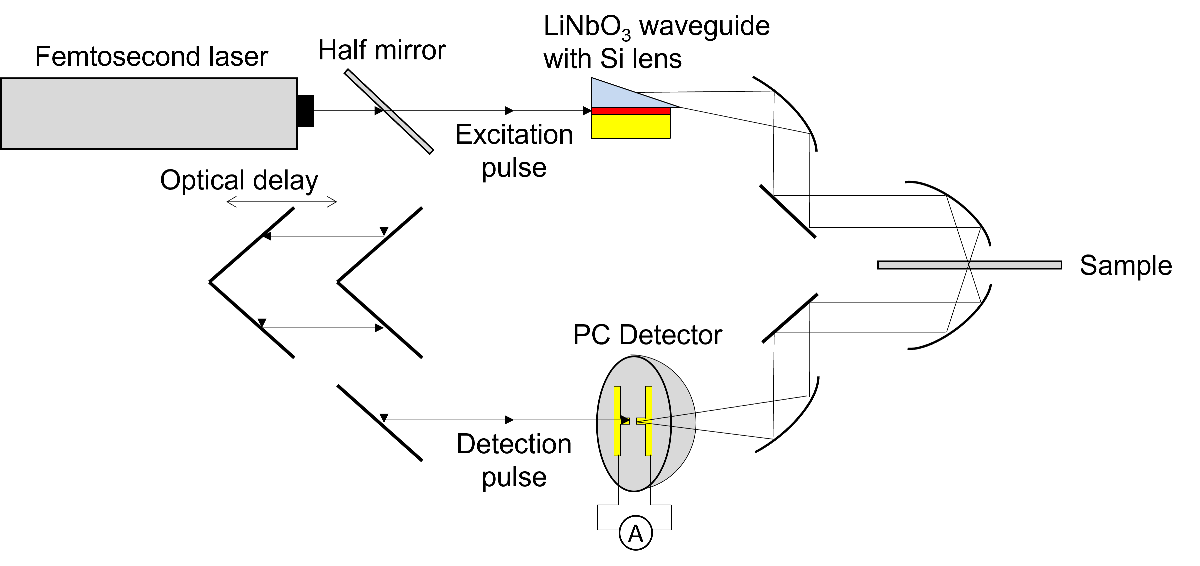


**Fig. S4:** Transient response of the photo–thermo–electric (PTE) sensor to pulsed light. When the pulse width of the incident wave is considerably smaller than the time constant of the sensor, the pulse wave can be regarded as a continuous wave emitter whose output is time-averaged. Therefore, the transient response to pulsed light is determined only by the time constant of the PTE sensor.


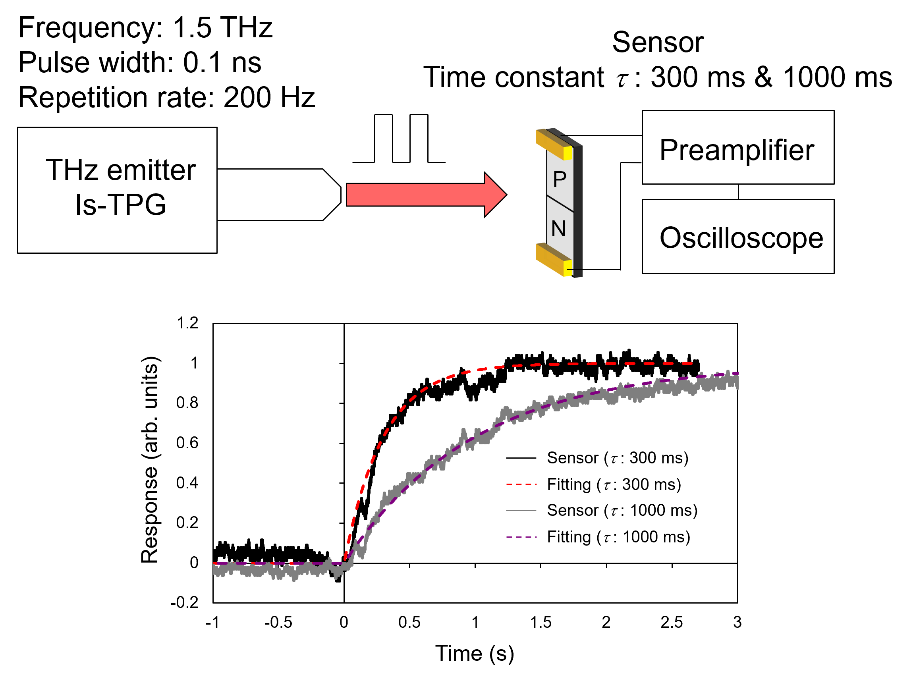


**Fig. S5:** Steady-state thermal distribution of film thickness. The distribution was calculated using the ANSYS software with the following simulation model: power and spot diameter of heat flow were 1 mW and 500 μm, respectively; the length, width, and thickness of the polyimide film were 5 mm, 3 mm, and 5 μm, respectively; the length, width, and thickness of the CNT film were 3 mm, 500 μm, and 0.1–5 μm, respectively. Images at the bottom row are examples of the thermal distribution, and the top graph is a summary of the simulated data.


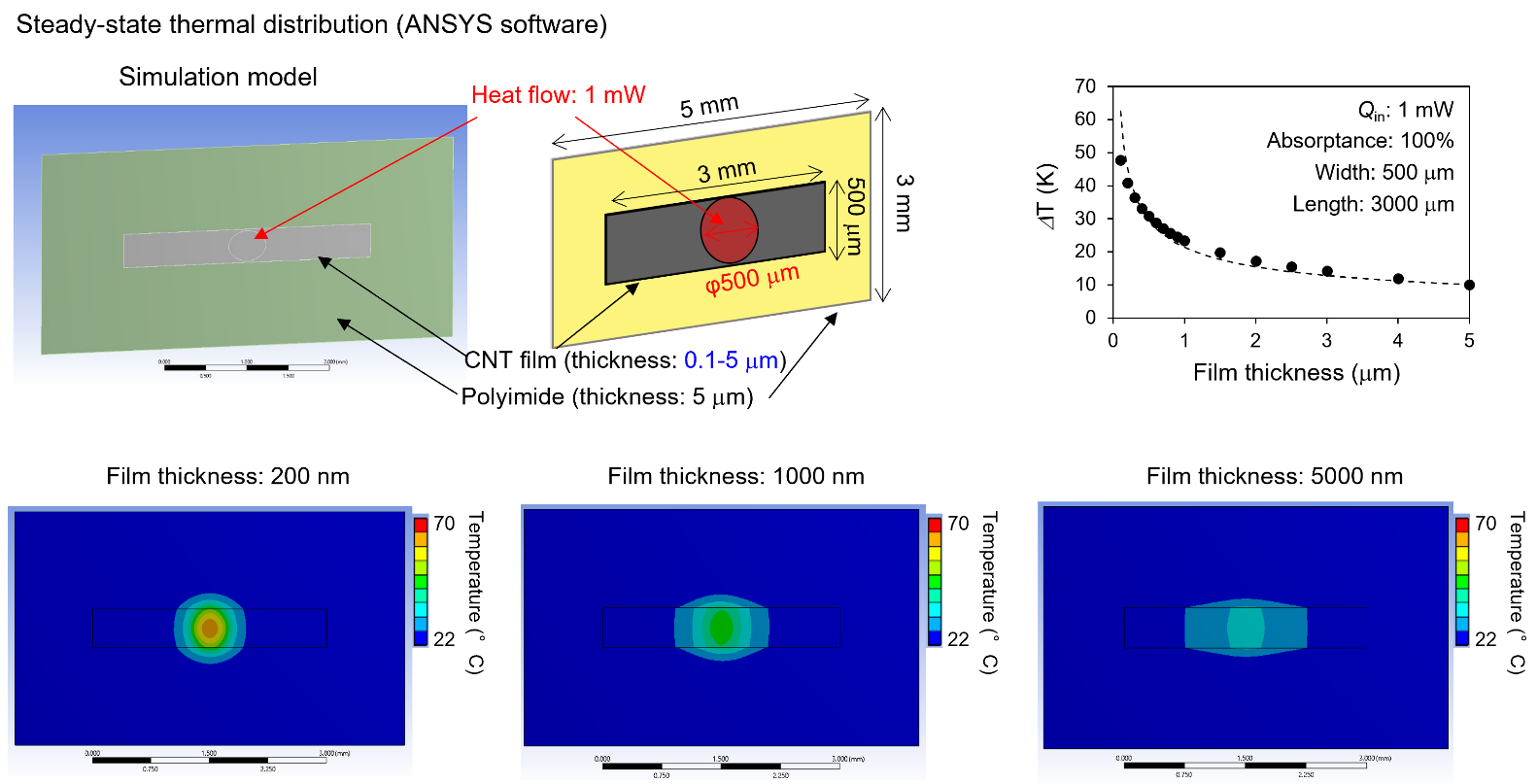


**Fig. S6:** Steady-state thermal distribution of film width. The distribution was calculated using the ANSYS software with the following simulation model: power and spot diameter of heat flow were 100 μW and 1 μm, respectively; the length, width, and thickness of the polyimide film were 5 mm, 3 mm, and 5 μm, respectively; the length, width, and thickness of the CNT film were 3 mm, 2–500 μm, and 3 μm, respectively. Bottom images are examples of the thermal distribution, and the top graph is a summary of the simulated data.


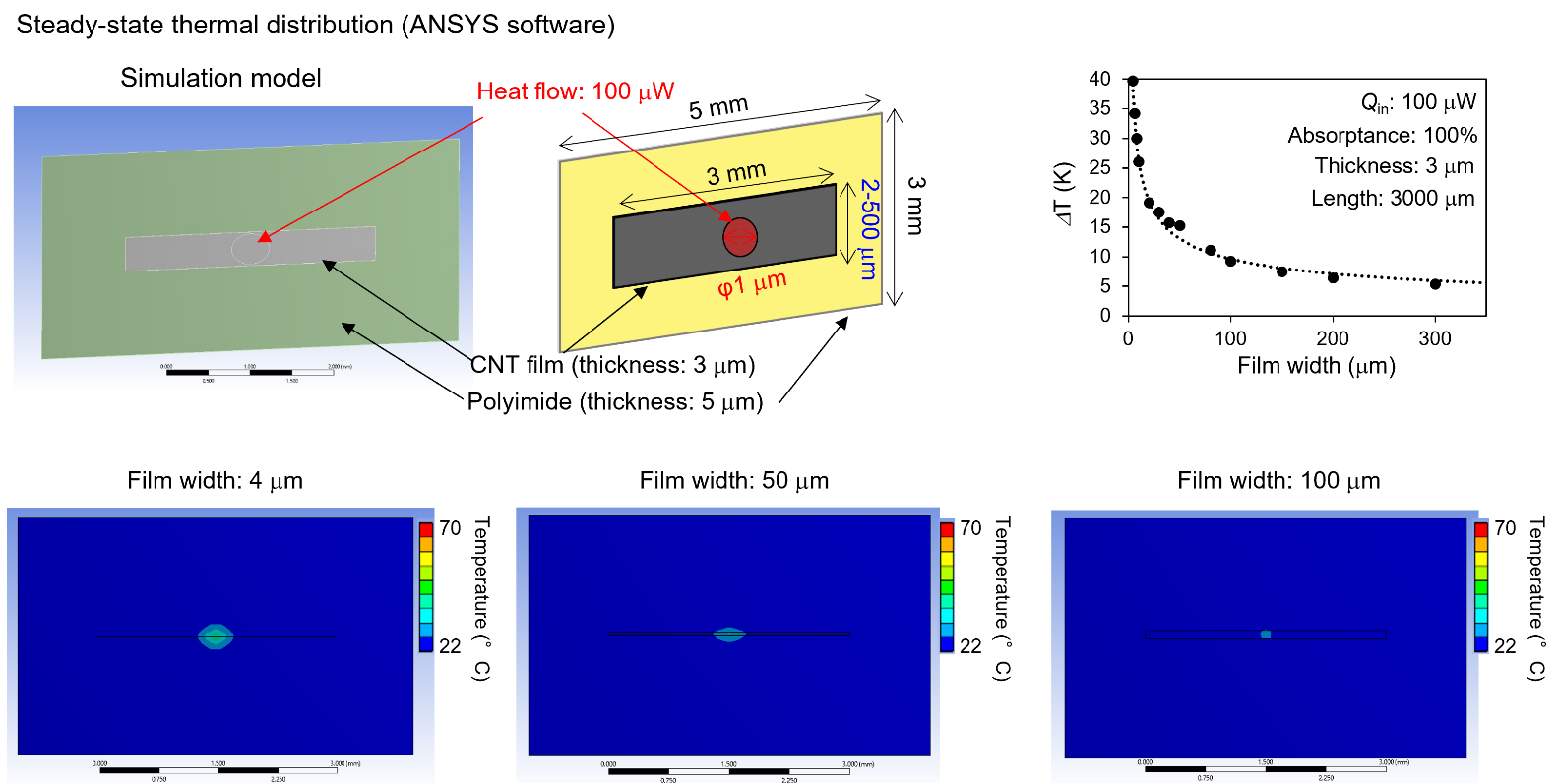


**Fig. S7:** Calculation method of THz sensitivity. (a) Schematics of the experimental system and total/reception THz power; (b) THz response dependence on film width; (c) Beam profile of the THz emitter; (d) Receiving intensity as a function of film width; (e) THz sensitivity dependence on film width.

In the film width optimization experiment, it is necessary to normalize the receiving THz power at each film width to calculate the sensitivity (voltage/receiving power) given that the light receiving area for THz light changes when the film width is changed. For this purpose, we first measured the beam profile of the incident light, wherein the full width at half maximum (FWHM) was 600 mm, as shown in Fig. S7c. According to this beam profile, the receiving intensity at each film width changes as shown in Fig. S7d. Finally, the sensitivity at each film width (Fig. S7e) was calculated by normalizing the THz response to the total incident power (Fig. S7b) at the reception intensity (Fig. S7d).


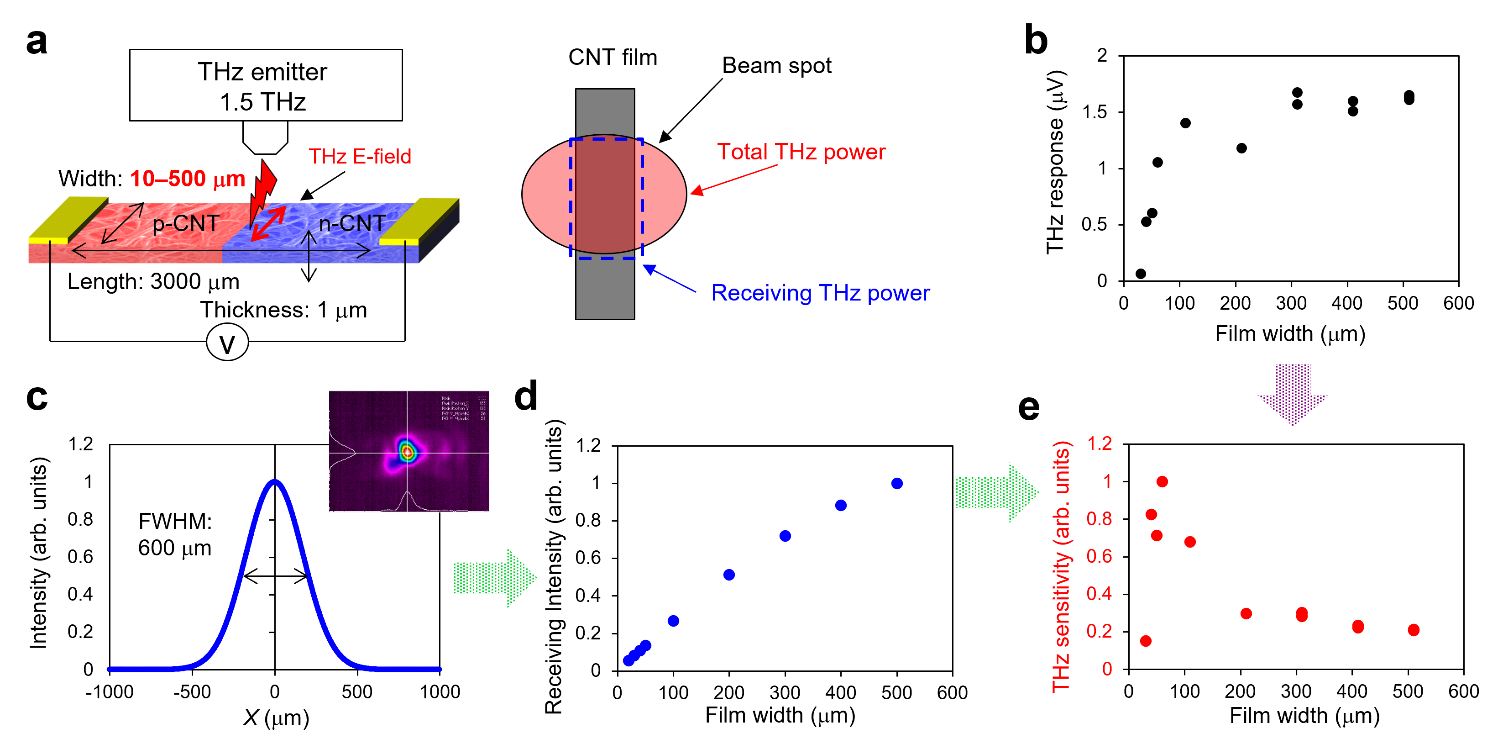


**Fig. S8:** (a) Schematic optical setup of THz-wave spectroscopy with the injection-seeded terahertz parametric generator (is-TPG). The is-TPG was driven by a sub-nanosecond pulsed laser at a repetition rate of 200 Hz. The THz waves were generated from the stimulated Raman scattering by phonon-polariton in a MgO:LiNbO_3_ crystal. The frequency of the THz wave was continuously tunable by tuning the wavelength of an injection seed CW laser. The THz-wave output from the is-TPG was focused onto a sample and then detected by a multi-element PTE sensor; (b) Power spectrum of the is-TPG measured by a multi-element PTE sensor.


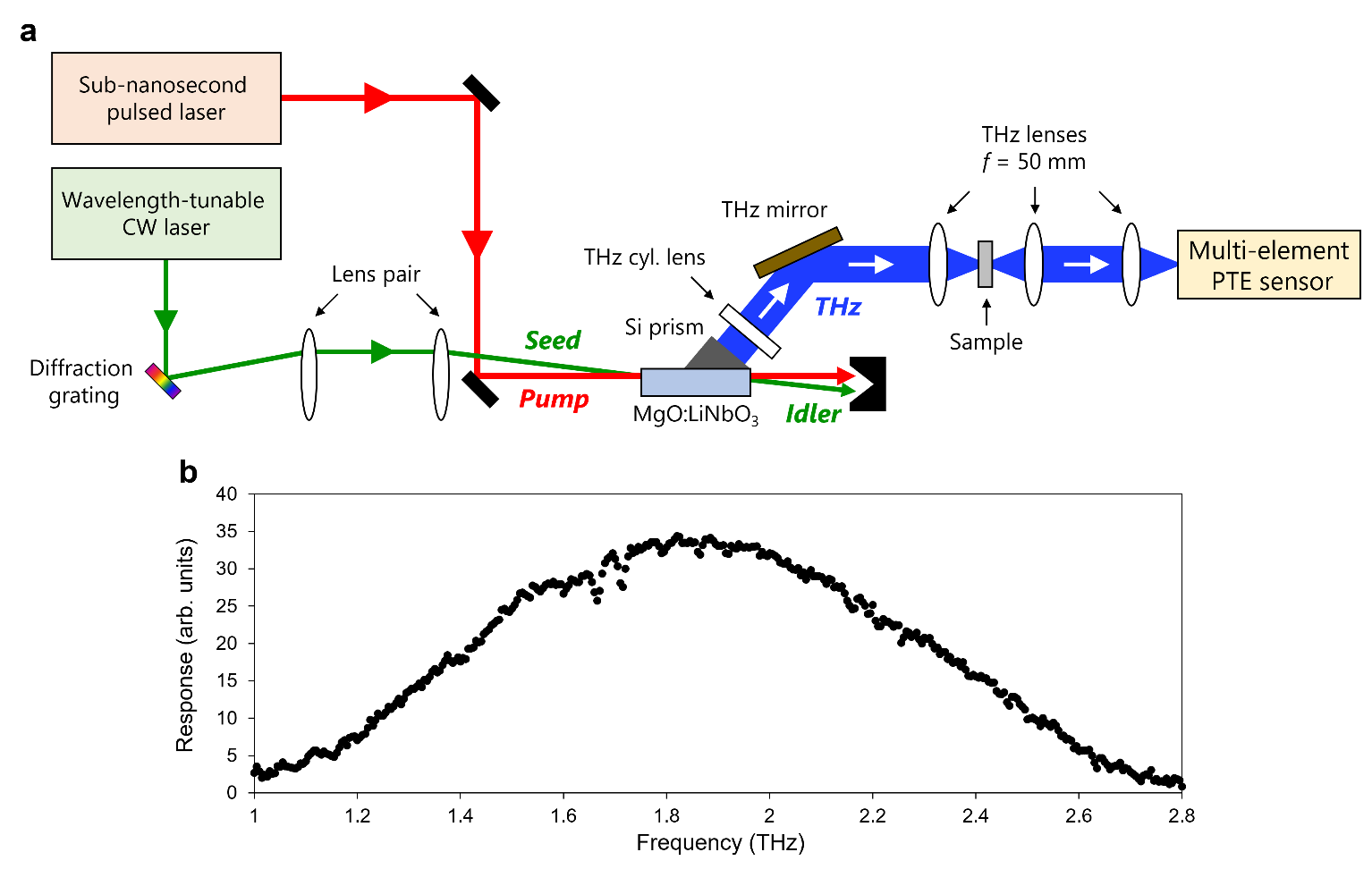

Supplement: Supplemental Material [file TSTA_A_2090855_SM8710.docx]
